# Vaccination: A New Research

**Published:** 1894-07-07

**Authors:** 


### The Hospital

An Institutional Journal of

The Medical Sciences and Hospital Administration, with

An Extra Nursing Supplement.

Vol. XVI.—No. 406.] [July 7, 1894.

### Vaccination: A New Research.

It is disappointing to lovers of assured scientific progress that, notwithstanding all the efforts which have been made during the last ten or twelve years to isolate the micro-organism which produces the peculiar phenomena of vaccinia, we seem to be as far off from any definite knowledge of the subject as we were at the commencement of the first research. Dr. Monckton Copeman publishes in the latest issue of the *Journal of Pathology and Bacteriology* an account of some additional researches made by him in this widely important field. We say "widely important," because the practice of vaccination is universal throughout the British dominions and the civilised world, and is everywhere forced upon native populations which come under European rule, almost at the point of the sword. In summarising his conclusions, Dr. Copeman announces as the first of them all the negative result that "the organisms to which the specific action of variolous and vaccine lymph is due are as yet undiscovered."

Now every member of the medical profession should take in this announcement in its full significance. At the present period of scientific progress, and especially in things medical, a rational scepticism is, of all tempers of mind, the most praiseworthy. Our medical ancestors have handed down to us certain results which seem to have stood the test of time and of an infinitely varied experience; and we are grateful to them. But our prime duty, even to those ancestors themselves, is to submit their results to the much more searching tests of our improved methods; to accept those, and those only, which come successfully out of our modern crucibles, and decisively to reject all the rest. This, one might think, would be the normal temper of mind among all men of scientific training, medical as well as others, and almost more than others. Unhappily for the progress of science, it is not so. Names count for as much in science and medicine as they do in politics and religion. But this is quite absurd. Names should be of no importance whatever where truth is concerned; and, as a matter of fact, they are of no importance except with the uncritical. Unfortunately, the uncritical constitute the vast majority, as well in medicine as in those professions which lay no claim to science at all.

It must be admitted, it cannot be denied, that the failure to isolate the organism of vaccinia and variola interposes a serious check upon scientific advancement in this direction. It is taken for granted by all explorers in these fields that there is such an organism, or at any rate that there are such organisms. Of course our conviction is that the organism of vaccinia, if there be one, must be identical in its essential characteristics with the organism of variola, if there be one. For the full and complete justification of Jenner's method of prophylaxis, this much seems to be entirely essential. Or if it be not essential, it is manifest that fields of investigation are opened out before us, which, even in these days of surpassing novelty, are of a decidedly novel character. But when we have to face the fact that the most careful investigations of vaccinia and its rise and progress yield no separable or cultivable microbe of vaccinia, and the equally carefully conducted investigations of the rise and progress of variola yield no separable and cultivable microbe of variola, we cannot but be staggered. We must call a halt. The most elementary loyalty to science, to common sense, and to self-respect, compels us to pause and to make whatever efforts may be possible to frame a scientific hypothesis for what we believe to be the facts and results of Jennerism.

Every medical man who believes in vaccination believes in it because he believes it to be based upon facts, and upon facts proved by the most rigid of experimental tests. But though we may believe, and believe most fully in our facts, we cannot but feel, as scientific men, the imperative obligation of explaining our facts. A fact believed and trusted in may be quite sufficient for ordinary practice and for the uncultured mind, but to science it is questionable whether a fact should be admitted to be a fact at all until it can be scientifically explained. It may indeed be a fact, but it is not a scientific fact. Now here is the most unfortunate thing in the whole history of the prophylactic treatment of small pox, that Jennerism, with all the helps which modern bacteriology can give it, is still a purely empirical proceeding, having no basis whatever in explicable and demonstrable science.

It seems necessary to put these facts in a clear and uncompromising light, and that in the interests of vaccination, of the public well-being, and of the scientific reputation of the medical profession. It is not that we are anxious to diminish medical faith in vaccination or to detract from the merit of Jenner's work: far from it. But the first of all scientific obligations is that the followers of scientific methods should see exactly where they are, and admit the exact truth concerning themselves and their position. Jenner, working on lines of folk-medicine in a country district, brought into use a method of prophylaxis, which in our judgment has robbed small-pox of its terrors and delivered the world from one of the most deadly scourges known to ancient or modern times. But he did not do more than this. He did not, he could not, with the methods then at his command, bring to a convincing demonstration the whole process and rationale of his work. Advancing by methods which though experimental were purely empirical, he gave us an empirical result; and it is on the purely empirical results of his work that the vaccination of the present day, with its world-wide consequences, is based.

A practical question arises here. Ought we to continue to vaccinate if we cannot give a scientific why and wherefore for our work? This question may be answered by asking another, or several others. Ought a baker to bake bread, and his customers to eat it, if he cannot give a scientific demonstration of the process of fermentation which takes place in the dough? Ought a farmer to sow corn who is not able to explain the chemico-physiological process of the development of the radicle and the plumule? The truth is—and we are almost ashamed to put so elementary a propositon into the pages of a scientific journal—that facts and empiricism, arts and manufactures, all take precedence of science in the order of human development. Whole generations of men—the majority, indeed, of mankind—have lived and worked and died without the consciousness that science was so much as possible. Science, the knowledge and comprehension of things in their essences and causes, is the latest of all human developments. We must then do with vaccination as we do with opium. We ask of opium what is its history; what has it done in medicine? And the answer is that of all substances which mankind has yet discovered it is the most indispensable for the beneficent work of the relief of human pain. Therefore, though we still lack a complete demonstration of the therapeutic *modus operandi* of opium, we continue to use it, and shall probably go on doing so to the end of time. Similarly we ask of vaccination this question of questions, What has it done in the world? And the answer is, that it has defeated in open conflict one of the deadliest enemies of mankind, and has put us upon lines of investigation which may yet rob all pathogenic organisms of their terrors. If such should be the grand result, it is obvious that the mind of man, by how much it is delivered from ignoble bodily fears, by so much will it rise to greater and greater heights of boldness and intellectual achievement. This we can all see. But medical men, as the custodians of the science as well as of the art of healing, must constantly remember that vaccination and inoculation can never achieve these magnificent results until they are firmly established upon a basis of scientific reason as well as upon a foundation of solid and unchallengeable fact.

